# The first draft genome of feather grasses using SMRT sequencing and its implications in molecular studies of *Stipa*

**DOI:** 10.1038/s41598-021-94068-w

**Published:** 2021-07-28

**Authors:** Evgenii Baiakhmetov, Cervin Guyomar, Ekaterina Shelest, Marcin Nobis, Polina D. Gudkova

**Affiliations:** 1 Institute of Botany, Faculty of Biology, Jagiellonian University, Gronostajowa 3, 30-387, Kraków, Poland; 2 Research Laboratory ‘Herbarium’, National Research Tomsk State University, Lenin 36 Ave., Tomsk, 634050 Russia; 3 German Centre for Integrative Biodiversity Research (iDiv), Puschstrasse 4, 04103 Leipzig, Germany; 4 Institute for Genetics, Environment and Plant Protection (IGEPP), Agrocampus Ouest, INRAE, University of Rennes 1, 35650 Le Rheu, France; 5 Centre for Enzyme Innovation, University of Portsmouth, Portsmouth, PO1 2UP UK; 6 Department of Biology, Altai State University, Lenin 61 Ave., Barnaul, Russia 656049

**Keywords:** DNA sequencing, Next-generation sequencing, Sequence annotation, Plant sciences, Genome

## Abstract

The Eurasian plant *Stipa capillata* is the most widespread species within feather grasses*.* Many taxa of the genus are dominants in steppe plant communities and can be used for their classification and in studies related to climate change. Moreover, some species are of economic importance mainly as fodder plants and can be used for soil remediation processes. Although large-scale molecular data has begun to appear, there is still no complete or draft genome for any *Stipa* species. Thus, here we present a single-molecule long-read sequencing dataset generated using the Pacific Biosciences Sequel System. A draft genome of about 1004 Mb was obtained with a contig N50 length of 351 kb. Importantly, here we report 81,224 annotated protein-coding genes, present 77,614 perfect and 58 unique imperfect SSRs, reveal the putative allopolyploid nature of *S. capillata*, investigate the evolutionary history of the genus, demonstrate structural heteroplasmy of the chloroplast genome and announce for the first time the mitochondrial genome in *Stipa.* The assembled nuclear, mitochondrial and chloroplast genomes provide a significant source of genetic data for further works on phylogeny, hybridisation and population studies within *Stipa* and the grass family Poaceae.

## Introduction

In the year 2000, the *Arabidopsis thaliana* L. genome became the first plant genome to be completely sequenced and assembled^1^. Since then, many genomes from the plant kingdom have been sequenced, e.g. green algae^2,3^, bryophytes^4,5^, ferns^6^, gymnosperms^7,8^ and angiosperms^9,10^. In the grass family (Poaceae) the reference assemblies were primarily obtained for crops^11–13^ and model plants^14–16^. The advent of second-generation sequencing and the subsequent decreasing of the overall sequencing costs have enabled the determination of whole genome sequences in many non-model plant species^17–20^.

Recently, the 1KP project that was aiming to sequence 1,000 green plant transcriptomes^21–23^ has been followed by the 10KP project^24^. The later initiative intends to sequence complete genomes from more than 10,000 plants and protists. The project is supposed to be completed in 2023 and it presumes to provide family-level high-quality reference genomes, ideally with chromosome-scale assemblies. Nevertheless, the data at the level of genera may not be processed immediately^24^. In comparison to other kingdoms, plants have very large genomes^13,25,26^, high ploidy level^27^ and the abundance of repetitive sequences^28–30^. Currently, to face these issues, the third-generation sequencing has been applied. The so-called single-molecule real-time (SMRT) sequencing provided by Pacific Biosciences (PacBio)^31^ and nanopore sequencing by Oxford Nanopore Technologies^32^ afford a range of benefits, including exceptionally long-read lengths (20 kb or more), resolving extremely repetitive and GC-rich regions and direct variant phasing^32,33^.

In the fossil record *Stipa* L., or a close relative genus, is known from about 34 Mya of the upper Eocene^34,35^. For many decades, *Stipa* has been described as a genus with over 300 species common in steppe zones of Eurasia, North Africa, Australia and the Americas^36,37^. According to the recent studies based on both morphological and molecular data, the genus has been reduced and currently includes over 150 species geographically confined to Europe, Asia and North Africa^38–42^. Most species of *Stipa* are dominants and/or subdominants in steppe plant communities^43–45^ and can be used for their classification^46^. Moreover, some species are of economic importance mainly as pasture and fodder plants, especially in the early phases of vegetation^36,47^, they can be used for soil remediation processes^48,49^, in studies related to climate change^50–52^ and as ornamental plants (e.g. *S. capillata* L., *S. pulcherrima* K. Koch*, S. pennata* L.).

In recent years, large-scale molecular data began to appear for *Stipa*: *de novo* transcriptome assemblies of *S. purpurea* Griseb.^50,53^, *S. grandis* P. A. Smirn.^54^ and *S. lagascae* Roem. & Schult.^52^, whole chloroplast genomes for 19 taxa^57^ and raw genomic data available via the NCBI Sequence Read Archive (SRA) for *S. capillata*^58^ and *S. breviflora* Griseb.^59^. In addition, nucleolar organising regions (NORs) were sequenced for six *Stipa* taxa^60^. Nevertheless, no complete or draft genome assembly currently exists for any *Stipa* species. In order to fill this gap, here we aim to: (1) present for the first time a single-molecule long-read dataset (nuclear, mitochondrial and chloroplast genomes) generated using the SMRT sequencing on the PacBio Sequel platform; (2) demonstrate and discuss the potential usage of this data in further studies of *Stipa*.

**Figure 1:**
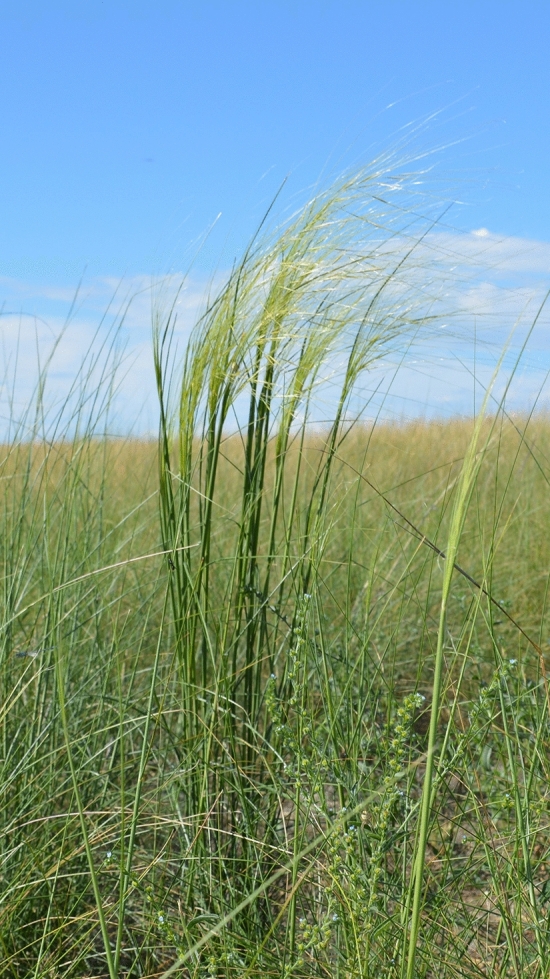
A representative individual of *Stipa capillata*.

For the goals of the study we chose to sequence the entire genome of *S. capillata* (Fig. 1) as it is the most widespread taxon within the genus, growing on sandy to loamy, nutrient poor soils in the dry grasslands of Eurasia^61^. Currently, this species is increasingly attracting the interest of conservation biologists due to its large distribution range, common occurrence in the Eurasian steppes and pseudosteppes, a limited number of refugia in Europe and both great morphological and genetic variability within its range^62–64^.

## Results

### Assembled nuclear genome

The SMRT sequencing yielded in 23.16-fold genome coverage consisting of 25.84 Gb sequence data with an N50 read length of 17,096 bp (Supplementary Table S1). *De novo* assembling of PacBio reads using Flye v.2.4^65,66^ resulted in a genome size of 1,004 Mb^67^ with a contig N50 of 351 kb and a GC level of 45.97%. On the other hand, another *de novo* assembly performed with FALCON v.0.2.5^68^ demonstrated a smaller genome size of 773 Mb with a GC level of 46.04%. However, the Flye assembly has a better N50 of 350,543 that is almost three times bigger than for FALCON. In case of applying Purge Haplotigs v1.1.1^69^, the final genome size was reduced by 177 Mb with an N50 of 381,155 (Table 1) and a GC level of 45.82%.

**Table 1:** Statistics of the nuclear genome assemblies.

Metrics	Flye assembly	FALCON assembly	Flye assembly after Purge Haplotigs
Length of assembly, bases	1,003,531,354	773,212,558	826,891,869
Number of sequences	5,931	885	3,683
Largest length of a sequence, bases	2,321,367	590,564	2,321,367
Average length of sequences, bases	169,201	88,015	224,516
N50, bases	350,543	119,836	381,155
Number of sequences with N50	837	2,061	640
N100, bases	1,001	20,078	1,014

The subsequent analysis based on a benchmark of 4,896 conserved genes belonging to the Poales order (dataset poales_odb10) revealed that the Flye assembly has 4,557 (93.10%) completed BUSCO (Benchmarking Universal Single-Copy) genes and only 293 (6%) missing BUSCOs versus 2,765 (56.50%) and 1,945 (39.70%) for the FALCON assembly. The Flye assembly after Purge Haplotigs shows 4,304 (87.90%) completed BUSCOs and 512 (10.50%) missing BUSCOs (Table 2).

**Table 2:** BUSCO statistics.

Metrics	Flye assembly	FALCON assembly	Flye assembly after Purge Haplotigs
Complete BUSCOs	4,557 (93.10%)	2,765 (56.50%)	4,304 (87.90%)
Complete and single-copy BUSCOs	2,383 (48.70%)	2,408 (49.20%)	2,916 (59.60%)
Complete and duplicated BUSCOs	2,174 (44.40%)	357 (7.30%)	1,388 (28.30%)
Fragmented BUSCOs	46 (0.90%)	186 (3.80%)	80 (1.60%)
Missing BUSCOs	293 (6%)	1,945 (39.70%)	512 (10.50%)
Total BUSCO groups searched	4,896 (100%)	4,896 (100%)	4,896 (100%)

### Scaffolding of contigs

Nearly all contigs of *S. capillata* genome can be assigned to the reference chromosomes of *Brachypodium distachyon* L., *Hordeum vulgare* L. and *Aegilops tauschii* Coss., whereas genomes of *Oryza sativa* L. and especially *Triticum aestivum* L., have much less homology to the feathergrass assembly. In particular, 95.16% contigs of *S. capillata* genome were assigned to seven chromosomes of *A. tauschii* genome, 94.68% to five chromosomes of *B. distachyon*, 94.20% to seven chromosomes of *H. vulgare*, 89.92% to 12 chromosomes of *O. sativa* and only 41.67% to 21 chromosomes of *T. aestivum.* The total length of non-assigned contigs was reasonably low for *A. tauschii* (48.59 Mb), *B. distachyon* (53.40 Mb) and *H. vulgare* (58.17 Mb), whereas for *O. sativa* and *T. aestivum* it was about 101.15 Mb and 585.40 Mb, respectively (Table 3). In addition, the RaGOO grouping confidence and orientation confidence scores per chromosome ranged from 57.81 to 76.11% and from 80.03 to 95.11%, respectively, indicating that the contigs could be placed on a chromosome with an acceptable level of confidence (Supplementary Table S2). The only exception is *T. aestivum* for which scores ranged from 30.49 to 47.76% for the grouping confidence score and from 57.81 to 70.19% for the orientation confidence score. Nevertheless, based on the location confidence score, the exact position of the contigs on a chromosome could not be accurately estimated, reflecting a low level of synteny to the reference genomes. In particular, the score was in a range of 31.30–43.66% for *O. sativa*, 26.06–39.13% for *B. distachyon*, 19.56–31.41% for *H. vulgare*, 17.47–24.15% for *A. tauschii* and 10.30–38.23% for *T. aestivum.*

**Table 3:** RaGOO statistics.

Species	Number of chromosomes (n)	Number and the total length of contigs assigned to the reference	Number and the total length of non-assigned contigs
*B. distachyon*^70^	5	4,061 (950.13 Mb)	1,871 (53.40 Mb)
		94.68%	5.32%
*H. vulgare*^71^	7	4,036 (945.36 Mb)	1,896 (58.17 Mb)
		94.20%	5.80%
*A. tauschii*^72^	7	4,161 (954.95 Mb)	1,771 (48.59 Mb)
		95.16%	4.84%
*O. sativa*^73^	12	3,477 (902.39 Mb)	2,455 (101.15 Mb)
		89.92%	10.08%
*T. aestivum*^74^	21	2,434 (418.14 Mb)	3,498 (585.40 Mb)
		41.67%	58.33%

### Transposable elements and nuclear genome annotation

Identification of transposable elements (TEs) revealed that more than half of the *S. capillata* genome (57.68%) is occupied by repetitive sequences. Particularly, retrotransposons represent at least 16.12% and transposons are reaching no less than 7.22% of the genome. Nonetheless, 34.34% of TEs are currently unclassified. Among classified repeats, long terminal repeats (LTRs) were the most abundant elements within retrotransposons, whereas Tourist/Harbinger elements were more common amid DNA-transposons. In total, 114,826 sequences were identified as simple repeats and occupy 0.57% of the genome. In addition, rolling-circles (0.28% of the genome) and low complexity sequences (0.11% of the genome) were found (Table 4).

**Table 4:** Statistics of repetitive elements.

Type of repeats	Number of elements	Total (bp)	% of genome
Class I: Retrotransposon:	123,524	161,756,598	16.12
SINEs	6,211	2,422,254	0.24
LINEs	26,453	19,189,619	1.91
LTR elements	90,860	140,144,725	13.97
Class II: DNA-transposon:	99,245	72,448,468	7.22
Hobo-Activator	6,824	3,826,368	0.38
Tc1-IS630-Pogo	619	500,988	0.05
PiggyBac	1	75	0.00
Tourist/Harbinger	11,326	3,980,231	0.40
Other	2	113	0.00
Unclassified	758,908	344,622,074	34.34
Total repeats	981,677	578,827,140	57.68
Rolling-circles	3,306	2,797,158	0.28
Low complexity	18,762	1,145,428	0.11
Simple repeats	114,826	5,716,291	0.57

The subsequent structural annotation of the masked genome revealed 53,535 nuclear genes (Supplementary File 1). On the other hand, the unmasked genome has 154,755 structurally annotated genes and 94,237 of them have BLAST hits in the NCBI non-redundant database. Nonetheless, among the 94,237 genes of the unmasked genome, 12,094 sequences are related to transposable elements. In particular, 2,925 genes associated with transposons, and 9,859 assigned to retrotransposons. In addition, 229 genes encode transposase-related proteins. Thus, except transposable elements the unmasked genome has 81,224 genes that can be associated with already known proteins (Supplementary File 2).

### SSR markers

In total, 77,614 perfect repeat motifs were identified for the nuclear genome assembly using Krait^75^ (Supplementary File 3). Within those, di- and tri-nucleotides were the most common types, accounting 28,365 (36.55%) and 25,794 (33.23%) repeats, respectively. Tetra-nucleotide motifs were the third most abundant repeats with 9,777 SSRs (12.60%), followed by mono-nucleotides with 6,572 SSRs (8.47%) and penta-nucleotides with 4,629 SSRs (5.96%). Hexa-nucleotides were the rarest motifs with 2,477 SSRs (3.19%). Only four mono-nucleotide, four di-nucleotide and three tetra-nucleotide motifs were found in the mitochondrial and chloroplast genomes. However, a total length of those SSRs was in a range of 12–16 bp. In addition, in total 58 unique repeats present only in a single copy in a range 101–325 bp were retrieved from the analysis of TEs. Within those were four hexa-, 35 hepta-, nine octa-, five nona- and five deca- nucleotide motifs (Supplementary Table S3).

### Divergence time of *Stipa*

**Figure 2:**
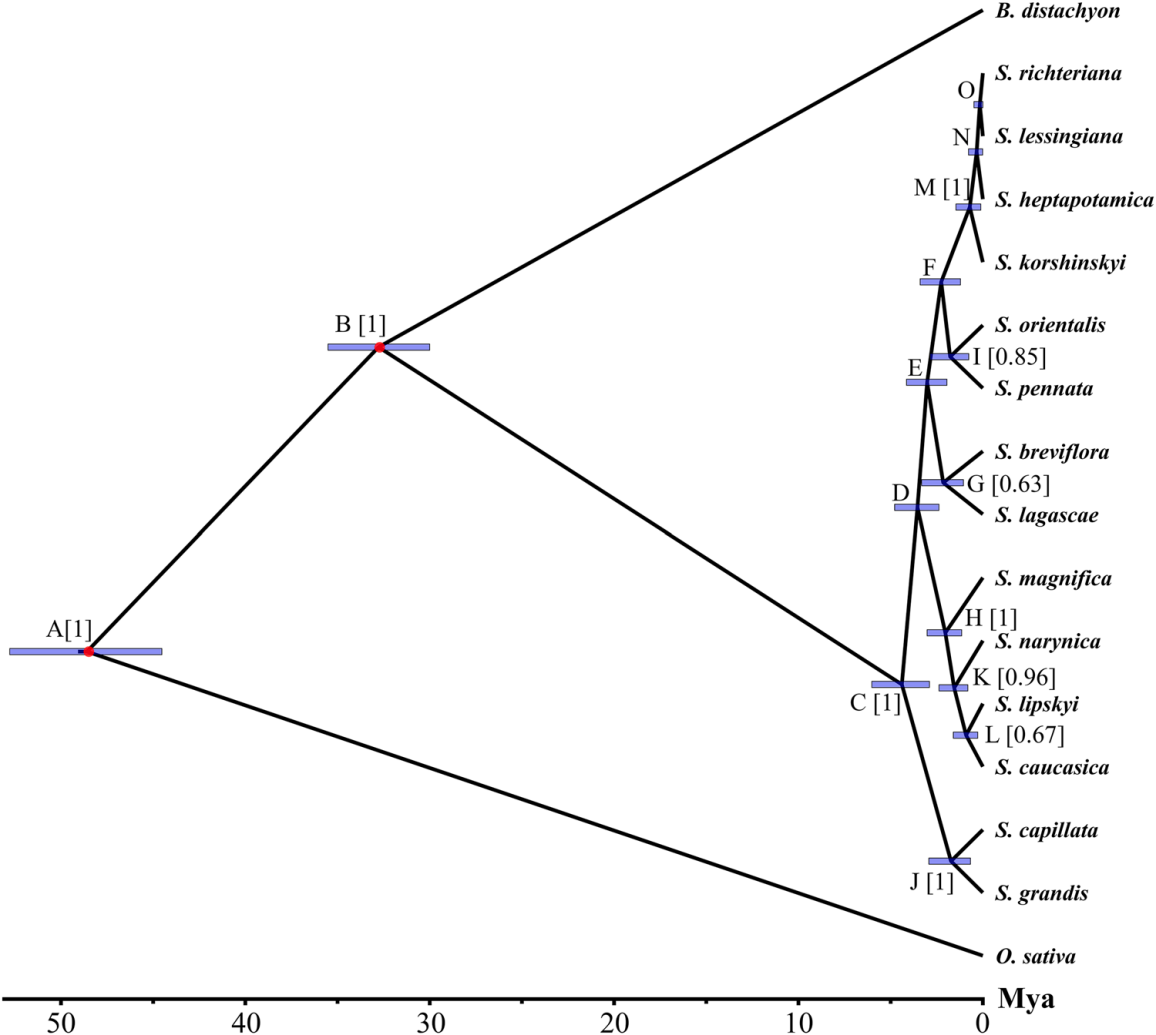
Phylogeny and divergence time estimation by molecular clock analysis. Letters at each node refer to Table 5. Numbers in brackets represent the Bayesian posterior probabilities (BPP > 0.50 only). The blue rectangles on the nodes indicate the 95% credibility intervals (CI) of the estimated posterior distributions of the divergence times. The red circles indicate the presumed divergence time splits set as a reference. The scale on the bottom shows divergence time in Mya. The figure was created using Figtree v1.4.4, https://tree.bio.ed.ac.uk/software/figtree/.

The Bayesian phylogenetic reconstruction based on the five loci within NORs revealed the divergence time of *Stipa* from *Brachypodium* around 30.00–35.52 Mya and the putative origin of feather grasses about 2.90–6.02 Mya (Fig. 2). Although not all branches were well supported within the genus, the current analysis confirmed the monophyly of *Stipa* and the general grouping of the analysed species regarding their taxonomic positions. In particular, *S. capillata* and *S. grandis* represent the section *Leiostipa* Dumort; *S. magnifica* Junge, *S. narynica* Nobis, *S. lipskyi* Roshev. and *S. caucasica* Schmalh. belong to the section *Smirnovia* Tzvelev. The remaining three groups include (1) *S. orientalis* Trin. and *S. pennata* L., (2) *S. richteriana* Kar. & Kir., *S. lessingiana* Trin. & Rupr., *S. heptapotamica* Golosk. and *S. korshinskyi* Roshev, (3) *S. lagascae* and *S. breviflora* currently have a discrepancy between morphological and molecular data. In addition, the divergence time estimation indicates that the potential origin of the clade comprising *S. capillata* and *S. grandis* is in a range of 0.67–2.93 Mya while the sister clade has the 95% credibility intervals for that parameter in a range of 2.38–4.78 Mya. Furthermore, the lowest genetic divergence time was registered for *S. lessingiana* and *S. richteriana* (0.00–0.48 Mya) as well as for the split between *S. heptapotamica* and the two above-mentioned species (0.01–0.78 Mya). The divergence times for the rest of taxa are present in Table 5.

**Table 5:** Node ages, BPP and CI related to Fig. 2.

Node	Node age (Mya)	BPP	95% CI
A	48.59	1.00	44.53–52.78
B	32.77	1.00	30.00–35.52
C	4.39	1.00	2.90–6.02
D	3.55	0.40	2.38–4.78
E	3.02	0.28	1.95–4.14
F	2.26	0.40	1.21–3.40
G	2.15	0.63	1.05–3.32
H	2.04	1.00	1.15–3.02
I	1.77	0.85	0.76–2.87
J	1.73	1.00	0.67–2.93
K	1.56	0.96	0.81–2.38
L	0.91	0.67	0.28–1.60
M	0.71	1.00	0.11–1.46
N	0.33	0.39	0.01–0.78
O	0.16	0.28	0.00–0.48

### Assembled mitochondrial and chloroplast genomes

**Figure 3:**
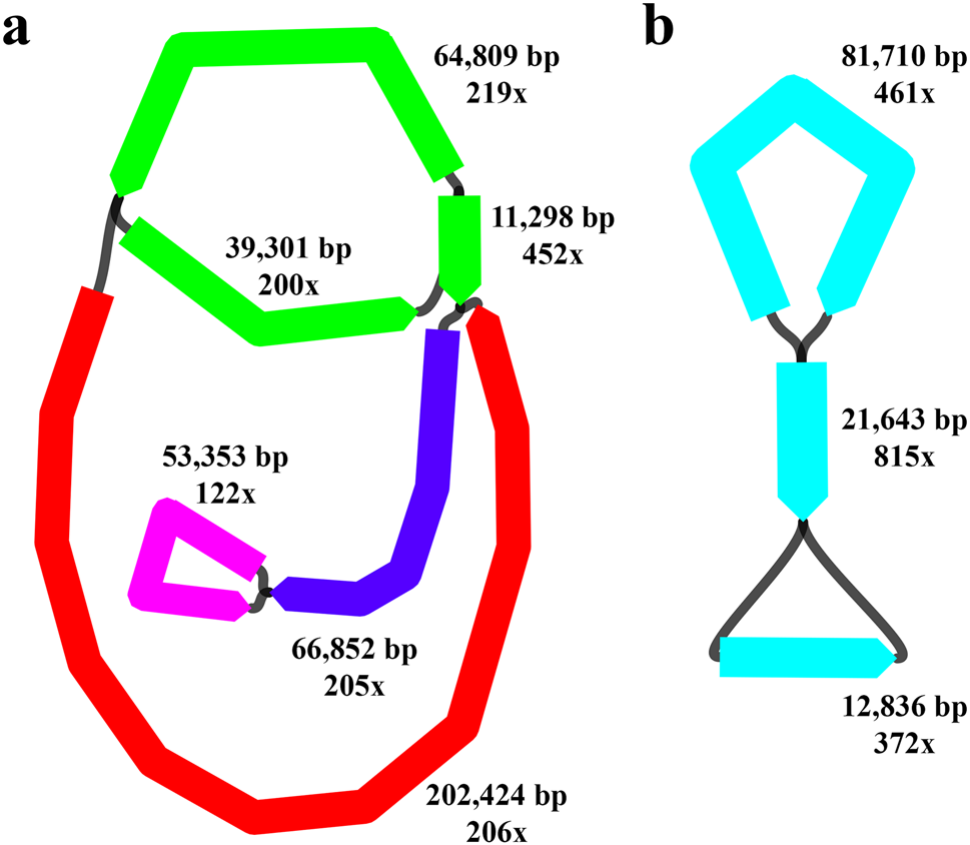
Visualisation of the *de novo* mitochondrial and chloroplast genome assemblies using Bandage v.0.8.1^85^. (**a**) Contigs representing mitochondrion. (**b**) Contig representing chloroplast. Different colours represent different contigs; length (in bp) and coverage (x) of edges within contigs are shown. The figure was created using Bandage v.0.8.1, https://rrwick.github.io/Bandage/.

The resulting Flye assembly contained four mitochondrial contigs with a total length of 438,037 bp^76–79^ represented by six edges and an entire 137,832 bp-long circular chloroplast genome combining a long single copy region (LSC) of 81,710 bp, a short single copy region (SSC) of 12,836 bp and two inverted repeats (IR) of 21,643 bp each (Fig. 3). However, after a manual checking in IGV v.2.8.6^80^ the final size of the chloroplast genome was slightly reduced to 137,823 bp. In addition, an analysis using Cp-hap^81^ detected two structural haplotypes of the chloroplast genome: haplotype A^82^ (LSC—IR, reverse-complement (rc)—SSCrc—IR) and haplotype B^83^ (LSC—IRrc—SSC—IR). We also obtained one assembly using Unicycler v.0.4.8^84^ resulted in 76 linear contigs from which 29 can be assigned to mitochondrial sequences with a total length of 1,668,569 bp. Due to the Unicycler assembly being more complex and none of the obtained contigs were likely to be circular in nature, for the downstream genome annotation we used the Flye assembly.

In total, 112 and 133 genes were functionally annotated for mitochondrial and chloroplast genomes, respectively. The mitochondrial annotation resulted in 78 protein-coding genes, 4 ribosomal RNA genes and 30 tRNA genes. The chloroplast annotation contained 85 protein-coding genes, 8 ribosomal RNA genes and 40 tRNA genes. The chloroplast genome size of 137,823 bp generated with Flye and the number of annotated genes in the current study were similar to the known assemblies for *S. capillata* obtained by Illumina sequencing^57^. However, the previous genome assemblies were slightly longer, specifically 137,830 bp^86^ and 137,835 bp^87^.

### DArTseq markers

The DArT pipeline analysis resulted in 61,328 Silico markers and in 52,970 sequences with SNPs. The BLAST process revealed 58,701 Silico markers and 52,252 sequences with SNPs that were successfully mapped to 4,361 and 3,935 genome contigs, respectively. Thus, the current genome assembly has 95.72% of Silico markers and 98.64% of sequences with SNPs that are represented in 73.52% (the total length of 969.30 Mb) and 66.34% (940.37 Mb) of the contigs, respectively. In addition, we established that 50,953 Silico markers and 47,181 sequences with SNPs were present only in a single copy in the genome. Finally, we identified 30 Silico markers and 10 sequences with SNPs aligned to the mitochondrial genome and only 2 Silico markers and 4 sequences with SNPs that were found in the chloroplast genome.

## Discussion

The number of sequenced plant genomes is rapidly increasing year by year serving as a fundamental resource for various genomic studies. In the current work, we present a 1004 Mb genome with the 23 × coverage of the most widespread feather grass species, *S. capillata*, using SMRT PacBio sequencing. The current assembly comprises 5,931 sequences with a contig N50 length of 351 kb (Table 1). The BUSCO completeness score of 93.10% (Table 2), the observation of a large portion of TEs (57.68%, Table 4) and the presence of Silico (95.72%) and SNPs (98.64%) markers derived from the DArT platform indicate that the assembly is of high quality. Moreover, the proportion of TEs has been reported for the first time in the genus due to the previous *de novo* assemblies which were performed exclusively based on transcriptomic data^50,52,54^. In addition, here we also attempted to perform a reference-guided scaffolding of the assembled contigs. Nevertheless, although nearly all contigs of the *S. capillata* genome were assigned to the chromosomes of *B. distachyon*, *H. vulgare* and *A. tauschii*, it was not possible to estimate their proper position on the reference with an acceptable level of confidence (Table 3 and Supplementary Table S2). In general, in the absence of a high-density genetic linkage map the task of reconstructing pseudomolecules of chromosomes seems to be challenging. On the other hand, we believe that in order to improve the contiguity of the long-read assembly the high-throughput chromosome conformation capture (Hi-C)^88^ technique should be applied. Currently, many studies on non-model species successfully utilised a combination of long-read techniques and Hi-C data to perform assemblies at chromosome scale^89–91^. Moreover, an additional key for improving this genome assembly in the future is merely to get more sequencing reads. Recently, it was shown that contig length metrics are positively correlated with both read length and sequence coverage. Specifically, long-read assemblies in maize demonstrated that the highest contig N50 of 24.54 Mb was reached with a subread N50 of 21,166 bp and a 75-fold depth of coverage while the longest contig of 79.68 Mb was observed with the same subread N50 but with a 60-fold depth^92^.

The newly generated genome has a GC content of 45.97% that is similar to the known estimates for species in *Stipa* varying in a range of 46.61–49.05%^93^, and more broadly to grasses ranging from 43.57% in *O. sativa* to 46.90% in *Z. mays*^94^. Recently, it was shown that a higher GC content in monocots is associated with adaptation to extremely cold and/or dry climates^95^. The genus *Stipa* highly supports this hypothesis due to the fact that all feather grasses are adapted to temperate, dry climates^36^. In addition, a positive correlation between the GC content and genome size was established^96^ suggesting insertion of LTR retrotransposons as a potential driving force of genome enlargement^97^. Similarly, here we showed that the expansion of the *S. capillata* genome also resulted from insertions of repetitive sequences that occupy 57.68% of the genome including LTR retrotransposons (13.97%). However, among all repetitive sequences around 34.34% are currently unclassified (Table 4). Nonetheless, the total proportion of TEs in *S. capillata* in comparison to other species within the Poaceae family is close to *Oryza minuta* J. Presl (58.35%) and *O. alta* Swallen^98^ (57.54%), bigger than in *B. distachyon*^99^ (28.10%) and *O. sativa*^100^ (45.52%) and smaller than in *O. granulata* Nees & Arn.^101^ (67.96%), *Avena sativa* L.^102^ (69.47%) and *T. aestivum*^103^ (84.67%).

Importantly, the presented genome size is roughly twice smaller than the expected size of 2,355 Mb and twice bigger than the expected monoploid size of 589 Mb estimated using flow cytometry^93^. Considering that we were unable to remove redundant sequences due to possible heterozygosity and the number of duplicated BUSCOs (Tables 1 and 2), it may be presumed that the current genome assembly combines two very distinct genomes. To the current knowledge, the vast majority of *Stipa* species have 44 (2n = 4x) chromosomes and are supposed to be tetraploids^41,104^. In addition, recently it was shown that a single-copy region *ACC1* and a low-copy nuclear gene *At103* have two different copies in *Stipa*^104,105^. Thus, it may suggest that *S. capillata*, and the genus *Stipa* in general, has arisen through hybridisation between genetically distant diploid species (2n = 22) and the subsequent allopolyploidisation via whole genome duplication (WGD) rather than via one WGD event of an ancestral species. Well-documented examples of natural allopolyploid taxa in the Pooideae subfamily are *Triticum turgidum* L. (2n = 4x = 28, genome constitution AABB) and *T. aestivum* (2n = 6x = 42, AABBDD) formed through hybridisation and successive chromosome doubling of ancestral diploid species *T. urartu* (2n = 2x = 14, AA), *Aegilops speltoides* Tausch. (2n = 2x = 14, BB) and *A. tauschii* (2n = 2x = 14, DD)^106^. Moreover, in the tribe Stipeae based on the *At103* gene allopolyploidy was reported for the genus *Patis* Ohwi (2n = 46, 48)^105^. Heretofore, at least three hypotheses were considered regarding the base chromosome number in Stipeae: x = 7^107^, x = 11^108,109^ and x = 12^110^. Recently, it was suggested that the latter two are more plausible^41,104^. Thus, in order to better assemble the *S. capillata* genome and verify if *Stipa* is an allopolyploid genus we suggest sequencing at chromosome level the close relative diploid species (2n = 22) from genera representing, e.g. *Ptilagrostis* Griseb., *Achnatherum* P. Beauv., e.g. *A. calamagrostis* L. (2n = 22 + 0‒2B), or *Piptatheropsis* Romasch., P. M. Peterson & Soreng (2n = 20, 22, 24)^41,104^.

In general, the number of genes in Poaceae varies from 28,835 in the smallest known genome, *Oropetium thomaeum* Trin. (2n = 20; genome size of 245 Mb)^111^, to 107,891 in *T. aestivum* (2n = 42; 14,547 Mb)^112^. Here, we reported 53,535 nuclear genes that were structurally annotated for the masked genome assembly. Such a number of genes was roughly 1.8 and 1.6 times smaller than previously determined for *S. grandis* (94,674 genes)^54^ and *S. purpurea* (84,298 genes)^50^, respectively. On the other hand, the annotation analysis of the unmasked genome resulted in 81,224 genes associated with already known proteins. In comparison, only 65,047 functionally annotated genes were reported for *S. grandis* while *S. purpurea* had 58,966. Nonetheless, as RNA-seq data is currently unavailable for *S. capillata*, we believe that the current version of the genome annotation demands a further investigation to properly characterise the genes sets when the appropriate information will be available.

SSR markers are widely distributed across the genome and they are commonly applied in establishing genetic structure in *Stipa*. Previously, polymorphic microsatellite primers were reported in populations of *S. purpurea* (11^113^, 15^114^ and 29^115^ loci), *S. pennata* (7 loci^116^), *S. breviflora* (21 loci^117^) and *S. glareosa* (9 loci^118^). In the present study, we identified 77,614 perfect SSR markers (Supplementary File 3) and 58 imperfect repeat motifs presented only in a single copy (Supplementary Table S3). Although we did not test them on the population level we are confident that such a number of new loci will be a valuable source for the farther development of SSR markers in *S. capillata*, and more generally in the genus *Stipa*. Additionally, the revealed loci could be used for the designing dominant inter simple sequence repeat (ISSR) markers^119^. Recently, the usefulness of applying ISSRs were shown for studies in *S. bungeana*^120^, *S. ucrainica* and *S. zalesskii*^121^, *S. tenacissima*^122^ and the hybrid complex *S. heptapotamica*^123^.

According to the previous studies, based on three chloroplast loci^124^ and four chloroplast loci and one nuclear region^105^, it was shown that the origin of Stipeae can be estimated in a range of 30.60–47.30 Mya and 21.20–39 Mya, respectively. Here, based on the five loci within NORs we demonstrated that the potential split between *Stipa* representing the tribe Stipeae and *Brachypodium* (the tribe Brachypodieae) took place approximately 30–35.52 Mya that supports the previous findings^105,124,125^. The present results also suggest that the genus *Stipa* likely originated ca. 4.39 (2.90–6.02) Mya. On the other hand, one previous study indicated the origin of feather grasses at about 12.90 Mya^124^ while another one showed different estimates based on chloroplast loci (21.20 Mya, 13–22) and the *At103* region^105^. Specifically, two copies of *At103* had the following suggested ages: 15.78 (6.30–26.60) Mya for the Eurasian Stipeae lineage and 5.62 (0–6.50) Mya for the American Stipeae lineage^105^. Thus, the latter estimate is close enough to the origin-age calculated in the current study. In addition, our data on the divergence time among *S. richteriana*, *S. lessingiana* and *S. heptapotamica* (Fig. 2 and Table 5) conforms to the previous findings on the ongoing hybridisation among these taxa^123^ suggesting NORs as a useful tool for revealing species of putative hybrid origin. Nonetheless, we believe that the current and previous estimates regarding the origin of *Stipa* should be treated with caution. Firstly, to our knowledge, there is still no available fossil data for any *Stipa* species from the Old World that can properly calibrate the historical diversification in the genus. Currently, the earliest definite *Stipa* caryopses were found in central Poland and are dated ca. 4,000 BC^126^. Secondly, available data demonstrate incongruence between chloroplast and nuclear loci analyses. In further studies we suggest utilising single-copy nuclear genes derived from whole genome sequencing projects. Thirdly, different sets of species and parameters used for inferring diversification dates may result in different estimates^127^.

Finally, we report a 137,823 bp chloroplast genome that is similar to the known assemblies in *Stipa* and specifically in *S. capillata*^57^. Here we highlight the applicability of a long-read sequencing technology like PacBio for the straightforward assembling of plastomes using Flye^67,68^. In addition, due to the long-reads we were able to identify two haplotypes presented in *S. capillata.* This result supports the previous findings in Poaceae^81^ suggesting that plastome structural heteroplasmy can be a common state in feather grasses. Moreover, for the first time in the genus *Stipa*, here we present a 438,037 bp mitochondrial genome. The current size of this genome is close to *Alloteropsis semialata* (R.Br.) Hitchc. (442,063 bp)^128^, *T. aestivum* (452,526 bp)^129^, *Sorghum bicolor* L. (468,628 bp)^130^ and *A. speltoides* (476,091 bp)^131^. Nevertheless, the present version of the genome is constituted by four contigs rather than one circular sequence. Although the general acceptance among mitochondrial biologists is that plant mitochondrial genomes have a variety of configurations^132–134^, in order to verify if a more accurate assembly could be performed, we suggest reusing our data for a more comprehensive analysis of the mitochondrial structures within *Stipa*.

## Materials and methods

### Plant material and DNA extraction

Our research complies with relevant institutional, national, and international guidelines and legislation. A *S. capillata* sample from Kochkor River Valley, central Kyrgyzstan (Supplementary Table S4), was selected for genome sequencing. The sample was stored in silica gel at ambient temperature until DNA extraction was performed. Total genomic DNA was isolated from dried leaves after a six-month storage period using a CTAB large-scale DNA extraction protocol (Supplementary information S1, described in Supplementary File 6). DNA extraction was performed by SNPsaurus (USA). In addition, we isolated DNA from dried leaves using a Genomic Mini AX Plant Kit (A&A Biotechnology, Poland). Subsequently, quality check, quantification and concentration adjustment were accomplished using a NanoDrop One (Thermo Scientific, USA) and agarose gel electrophoresis visualisation. The concentration of the sample was adjusted to 50 ng/μL. The purified DNA sample (1 μg) was sent to Diversity Arrays Technology Pty Ltd (Canberra, Australia) for sequencing and DArT marker identification. Moreover, to test the phylogenetic power of NORs in *Stipa*, we supplemented the study with five specimens of *S. richteriana* Kar. & Kir, three of *S. lessingiana* Trin. & Rupr., four of *S. heptapotamica* Golosk. and four of *S. korshinskyi* Roshev. (Supplementary Table S4). The isolation of genomic DNA was performed from dried leaf tissues using a modified CTAB method^135^.

### Library construction and sequencing

In total, 5 ug of *S. capillata* genomic DNA were used to construct a PacBio library according to the 20 kb PacBio template preparation protocol omitting a shearing step. The size selection cut-off was set at 15 kb. The library preparation followed by sequencing on three PacBio Sequel SMRT cells (Pacific Biosciences, Menlo Park, CA, USA) was carried out by SNPsaurus, LLC. Prior to the assembly, reads from each SMRT cell were inspected and quality metrics were calculated using SequelQC v.1.1.0^136^. A high-density assay using the DArT complexity reduction method for *S. capillata* was performed according to a previously reported procedure^137^.

For the rest of the specimens used in the current study, the quality control using a fluorometer (PerkinElmer Victor3, USA) and gel electrophoresis, library construction using a TruSeq Nano DNA Library kit (350 bp insert size; Illumina, USA) and sequencing using 100 bp paired-end reads on an Illumina HiSeq 2500 platform (Illumina, USA) were performed by Macrogen Inc. (South Korea).

### Nuclear genome assembly and validation

The execution of this work involved using many software tools, whose versions, settings and parameters are described in Supplementary information S2 (available in Supplementary File 6). The *de novo* assembly of the PacBio data was performed using Flye v.2.4^65,66^. The draft assembly was cleaned by running BLASTn v.2.10.0^138^ against the NCBI nucleotide database v.5, and subsequently sending each BLAST hit to the JGI taxonomy server (https://taxonomy.jgi-psf.org/) with a downstream step of keeping only plant contigs. Thereafter, Qualimap v.2.2.2^139^ was used to identify mean coverage for each contig. In the final assembly we kept only contigs with an average coverage of more than 10x. In addition, overrepresented contigs (> 60x) were BLASTed against the NCBI nucleotide database v.5 and sequences assigned to chloroplasts and mitochondria were removed.

Due to the final assembly performed with Flye v.2.4 being roughly twice bigger than an expected monoploid genome size of 589 Mb^93^, we accomplished an additional assembly with FALCON v.0.2.5^68^ and applied Purge Haplotigs v1.1.1^69^ to filter redundant sequences due to possible heterozygosity. The assemblies' statistics were analysed using assembly-stats v.1.0.1^140^. In addition, in order to assess the completeness of the genome assemblies, we investigated the presence of highly conserved orthologous genes using BUSCO v.4.0.6^141^.

### Scaffolding of contigs

Due to there being no reference genome for any *Stipa* species, here we applied RaGOO v.1.1^142^ to verify if a reference-guided scaffolding can be performed for the draft genome contigs based on four genomes from the Pooideae subfamily (*B. distachyon*^70^, *H. vulgare*^71^, *A. tauschii*^72^, *T. aestivum*^74^) and one genome from the Oryzoideae subfamily (*O. sativa*^73^). The subsequent assessment of the scaffolding accuracy was based on three parameters: (1) location confidence score, (2) orientation confidence score and (3) grouping confidence score^142^.

### Repeat prediction and nuclear genome annotation

The repeat prediction for *S. capillata* was performed using a *de novo* transposable element (TE) family identification and modeling package RepeatModeler v.2.0.1^143^ which includes three repeat finding programs; RECON^144^, RepeatScout^145^, and TRF^146^. The resulting TE library was supplemented by the transposable elements database (Release 19, http://botserv2.uzh.ch/kelldata/trep-db/).^147^ Subsequently, the genome assembly was masked for TEs regions by RepeatMasker v.4.1.0^148^ (http://repeatmasker.org) with the search engine RMBlast v.2.9.0 + ^149^ and the custom library created in the previous step. Next, gene and protein sequences were predicted using Augustus v.3.2.3 with the unmasked and v.3.3.3^150^ with the masked genome assemblies. The predicted protein sequences of the unmasked assembly were then BLASTed against the NCBI protein database v.5 and the subsequent BLAST hit descriptions were added to GFF (General Feature Format) files.

### Genome-wide identification of microsatellite markers

The unmasked nuclear genome, chloroplast and mitochondrial genome assemblies were screened for perfect mono-, di-, tri-, tetra-, penta- and hexa-nucleotide repeat motifs using Krait v.1.3.3^75^. We applied the following criteria: mono-nucleotide repeat motifs contain at least 12 repeats, di-nucleotide repeat motifs contain at least seven repeats, tri-nucleotide repeat motifs contain at least five repeats, tetra-, penta- and hexa-nucleotide repeat motifs contain at least four repeats.

### Divergence time of *Stipa*

In order to estimate the divergence between *S. capillata* and other *Stipa* species we used the nucleolar organising regions. Firstly, we prepared a set of reference sequences including *S. lipskyi* Roshev.^151^, *S. magnifica* Junge^152^, *S. narynica* Nobis^153^, *S. caucasica* Schmalh.^154^, *S. orientalis* Trin.^155^ and *S. pennata* L.^156^. Secondly, we mapped raw reads of *S. capillata*, *S. richteriana*, *S. lessingiana*, *S. heptapotamica* and *S. korshinskyi* (Supplementary Table S2) as well as *S. grandis*^55^, *S. breviflora*^59^, *S. lagascae*^157^ to the reference set using Minimap2 v.2.17-r941^158^ with keeping only uniquely mapped reads by Samtools v.1.9^159^. Thirdly, the *de novo* assembly of the NORs was performed using Canu v.2.0^160^ for *S. capillata* and SPAdes v.3.14.1^161^ for the rest of *Stipa* species. Additionally, we added to the analysis *B. distachyon*^162^ as an ingroup member of the Pooideae subfamily and *O. sativa*^163^ as an outgroup representing the Oryzoideae subfamily within the Poaceae family. Next, all sequences were aligned using MAFFT v.7.471^164^. Subsequently, the aligned sequences were visualised in AliView v.1.26^165^ and divided in five loci: (1) 18S ribosomal RNA, (2) Internal Transcribed Spacer 1 (ITS1), (3) 5.8S ribosomal RNA, (4) Internal Transcribed Spacer 2 (ITS2) and (5) 26S ribosomal RNA (Supplementary File 4). Estimation of divergence times was performed in BEAST2 v.2.6.3^166^ using the 121,321 substitution model determined by bModelTest^167^. We used the following constraints for time calibrations: 38–48 million years ago (Mya) for the *Brachypodium*-*Oryza* split^101^ and 33–39 Mya for the potential origin and divergence of *Stipa*^34,35^. Then, the divergence time was estimated using the strict clock model and the Yule prior. In total, we ran the analysis three times independently, 50 million Markov chain Monte Carlo (MCMC) generations for each run. The log and tree files were combined using LogCombiner v.2.6.3 (a part of the BEAST package) with the first five million generations discarded as burn-in from each run. Next, Tracer v.1.7.1^168^ was used to check the log files regarding Effective Sample Size (ESS) values. As all ESSs exceeded 200, we summarised the final maximum clade credibility tree (Supplementary File 5) in TreeAnnotator v.2.6.3 (a part of the BEAST package). The final tree was visualised and edited using FigTree v.1.4.4^169^.

### Mitochondrial and chloroplast genomes assembly, annotation and validation

Prior to assembly, we mapped raw reads to 11 reference mitochondrial genomes of species belonging to the Poaceae family (Supplementary Table S5) using Minimap2 v.2.17-r941^158^. Only uniquely mapped reads were kept by Samtools v.1.9^159^ for the next step. *De novo* mitochondrial assembly of the 4.08 Mb data was performed using Flye v.2.7.1-b1590.

In the next step, we BLASTed the resulting contigs against the NCBI nucleotide database v.5, and sequences assigned to mitochondria were kept. Then, the PacBio subreads were mapped onto the kept contigs using Minimap2, and only uniquely mapped reads were retained by Samtools. A new *de novo* assembly of the 15.51 Mb data was performed using Flye. In order to check if the mitochondrial contigs obtained by Flye could be merged into larger scaffolds we applied Circlator v.1.5.5^170^. However, the resulting sequences were identical to the Flye contigs. In addition, we used Unicycler v.0.4.8^84^ with reads that were mapped onto the Flye contigs as a reference.

Further, to detect all possible structural haplotypes of the chloroplast genome we applied Cp-hap^81^. Next, we mapped raw reads onto the resulting mitochondrial contigs and the chloroplast genomes to manually check in IGV v.2.8.6^80^ if any potential SNPs or indels are present. Eventually, annotations of the final mitochondrial contigs of 438,037 bp and the chloroplast genomes of 137,823 bp were performed using Geneious Prime v.2021.1.1 (https://www.geneious.com) based on 85% and 95% similarities to the reference genomes of mitochondria and chloroplasts, respectively (Supplementary Table S5).

### In Silico mapping of DArT marker sequences

Since the DArT markers are designed to target active regions of the genome^171^, here we use them to validate the completeness of the nuclear genome assembly and improve the accuracy of data filtering in further genomic studies on *Stipa*. Two data types, Silico and SNPs markers, were mapped to the nuclear genome using BLASTn v.2.10.0. As a query we used trimmed DArT sequences in a range of 29–69 bp with the percent identity values to the reference genome of 95% or greater and removing alignments below 95% of a query.

## Supplementary Information

Supplementary File 1.

Supplementary File 2.

Supplementary File 3.

Supplementary File 4.

Supplementary File 5.

Supplementary File 6.

## Data Availability

The raw PacBio reads are available at NCBI Sequence Read Archive^172^. The final genome assemblies are deposited into NCBI Assembly database under the following Accession Numbers: nuclear assembly (JAGXJF000000000)^67^; mitochondrion assembly, contig 1 (MZ161090)^76^, contig 2 (MZ161091)^77^, contig 3 (MZ161093)^78^ and contig 4 (MZ161092)^79^; chloroplast assemblies, haplotype A (MZ146999)^82^ and haplotype B (MZ145043)^83^. The masked and the unmasked versions of the nuclear genome annotation are presented in the Supplementary File 1 and the Supplementary File 2, respectively.
