# Supplementary material for: The first draft genome of feather grasses using SMRT sequencing and its implications in molecular studies of Stipa
Source: Sci Rep. 2021 Jul 28;11:15345. doi: 10.1038/s41598-021-94068-w (PMC8319324; doi:10.1038/s41598-021-94068-w)
Supplement: Supplementary file 6 [file 41598_2021_94068_MOESM6_ESM.docx]

The first draft genome of feather grasses using SMRT sequencing and its implications in molecular studies of *Stipa*

Evgenii Baiakhmetov^1,2*^, Cervin Guyomar^3,4^, Ekaterina Shelest^3,5^, Marcin Nobis^1,2*^, Polina D. Gudkova^2,6^

^1^ Institute of Botany, Faculty of Biology, Jagiellonian University, Gronostajowa 3, 30-387 Kraków, Poland

^2^ Research laboratory 'Herbarium', National Research Tomsk State University, Lenin 36 Ave., 634050 Tomsk, Russia

^3^ German Centre for Integrative Biodiversity Research (iDiv), Puschstrasse 4, 04103 Leipzig, Germany

^4^ IGEPP, Agrocampus Ouest, INRAE, University of Rennes 1, 35650 Le Rheu, France

^5^ Centre for Enzyme Innovation, University of Portsmouth, PO1 2UP Portsmouth, UK

^6^ Department of Biology, Altai State University, Lenin 61 Ave., 656049 Barnaul, Russia

*Corresponding Authors:

Evgenii Baiakhmetov^1,2^

Gronostajowa 3, Kraków, 30-387 Kraków, Poland

Email address: evgenii.baiakhmetov@doctoral.uj.edu.pl

Marcin Nobis^1,2^

Gronostajowa 3, Kraków, 30-387 Kraków, Poland

Email address: m.nobis@uj.edu.pl

Supplementary Table S1. Statistics for the PacBio long-reads dataset.

| Read type | Read bases | Read number | Read length (max) | Read length (mean) | Read length (N50) |
| --- | --- | --- | --- | --- | --- |
| Subreads, cell 1 | 9,748,472,200 | 907,975 | 170,673 | 10,736 | 17,383 |
| Subreads, cell 2 | 7,384,978,143 | 813,520 | 102,206 | 9,078 | 15,472 |
| Subreads, cell 3 | 8,706,147,385 | 785,976 | 205,974 | 11,077 | 17,542 |

Supplementary Table S2. Average RaGOO confidence scores for the draft genome of *S. capillata* assigned to chromosomes of the five reference species.

| **Chromosome** | **Reference species** | **No. of assigned contigs** | **Location confidence scores** | **Orientation confidence scores** | **Grouping confidence score** |
| --- | --- | --- | --- | --- | --- |
| 1 | *Brachypodium distachyon* (BD) | **1,312** | 26.06% | 84.59% | 73.23% |
|  | *Hordeum vulgare* (HV) | 519 | 22.53% | 82.46% | 59.78% |
|  | *Aegilops tauschii* (AT) | 528 | 24.15% | 84.81% | 57.81% |
|  | *Oryza sativa* (OS) | 467 | 34.22% | **92.80%** | **73.72%** |
|  | *Triticum aestivum* (TA) | 108 | **36.86%** | 90.34% | 42.60% |
| 2 | BD | **794** | 28.21% | 89.27% | **74.85%** |
|  | HV | 701 | 19.56% | 82.32% | 59.04% |
|  | AT | 728 | 17.47% | 80.03% | 57.87% |
|  | OS | 360 | **36.79%** | **91.82%** | 71.46% |
|  | TA | 76 | 34.91% | 91.03% | 43.09% |
| 3 | BD | **813** | 31.57% | 89.29% | 74.38% |
|  | HV | 558 | 21.66% | 85.59% | 63.04% |
|  | AT | 677 | 20.62% | 84.93% | 59.70% |
|  | OS | 344 | **33.68%** | **95.11%** | **76.11%** |
|  | TA | 90 | 22.81% | 88.91% | 34.18% |
| 4 | BD | **825** | 30.23% | 85.85% | 67.93% |
|  | HV | 649 | 31.41% | 85.27% | 59.75% |
|  | AT | 571 | 20.54% | 84.30% | 59.24% |
|  | OS | 299 | **37.13%** | **91.91%** | **69.08%** |
|  | TA | 106 | 30.50% | 87.09% | 37.79% |
| 5 | BD | 317 | 39.13% | 90.50% | **72.58%** |
|  | HV | 614 | 26.34% | 86.36% | 61.56% |
|  | AT | **664** | 19.44% | 83.30% | 60.63% |
|  | OS | 295 | **41.53%** | **92.83%** | 70.77% |
|  | TA | 112 | 38.23% | 89.43% | 43.97% |
| 6 | HV | 370 | 24.91% | 83.60% | 59.72% |
|  | AT | **398** | 23.54% | 82.26% | 58.25% |
|  | OS | 307 | **38.53%** | **91.13%** | **70.58%** |
|  | CR | 129 | 12.97% | 83.24% | 36.94% |
| 7 | HV | **625** | 23.24% | 82.34% | 60.17% |
|  | AT | 595 | 23.94% | 82.34% | 62.74% |
|  | OS | 235 | **37.73%** | **92.68%** | **72.96%** |
|  | TA | 96 | 25.65% | 90.27% | 40.23% |
| 8 | OS | **311** | **37.91%** | **91.85%** | **66.79%** |
|  | TA | 92 | 31.07% | 89.58% | 40.32% |
| 9 | OS | **210** | **39.61%** | **92.80%** | **70.22%** |
|  | TA | 147 | 30.99% | 87.64% | 37.79% |
| 10 | OS | **188** | **31.30%** | **92.97%** | **64.34%** |
|  | TA | 90 | 24.12% | 86.06% | 37.89% |
| 11 | OS | **287** | **35.98%** | 87.07% | **61.05%** |
|  | TA | 74 | 27.42% | **90.49%** | 39.98% |
| 12 | OS | **174** | **43.66%** | **91.85%** | **63.87%** |
|  | TA | 102 | 23.66% | 86.64% | 37.53% |
| 13 | TA | 104 | 25.17% | 85.16% | 44.78% |
| 14 | TA | 108 | 32.12% | 92.44% | 47.76% |
| 15 | TA | 113 | 18.34% | 88.77% | 36.13% |
| 16 | TA | 69 | 31.05% | 85.61% | 37.87% |
| 17 | TA | 82 | 27.67% | 87.67% | 40.31% |
| 18 | TA | 58 | 30.93% | 89.12% | 38.11% |
| 19 | TA | 239 | 17.54% | 78.84% | 36.22% |
| 20 | TA | 133 | 23.32% | 83.71% | 40.41% |
| 21 | TA | 306 | 10.30% | 70.19% | 30.49% |

Supplementary Table S3**.** Unique imperfect SSRs presented in the nuclear genome of *S. capillata***.**

| ID | Contig number | Motif | Type | Start | End | Length |
| --- | --- | --- | --- | --- | --- | --- |
| 1 | contig_907 | (TTGACA)n | hexa | 214,212 | 214,373 | 164 |
| 2 | contig_1647 | (TCTGGT)n | hexa | 65,778 | 65,902 | 128 |
| 3 | contig_4419 | (TATGTC)n | hexa | 22,183 | 22,302 | 122 |
| 4 | contig_3408 | (GGAATT)n | hexa | 106,465 | 106,560 | 102 |
| 5 | contig_232 | (TTCGGGG)n | hepta | 1,513,940 | 1,514,259 | 326 |
| 6 | contig_2603 | (TAGGGTC)n | hepta | 144,295 | 144,587 | 293 |
| 7 | contig_2186 | (GGCTTAG)n | hepta | 45,772 | 46,082 | 291 |
| 8 | contig_1032 | (ACCCCGG)n | hepta | 99 | 351 | 243 |
| 9 | contig_3460 | (CCGGCCC)n | hepta | 201,077 | 201,281 | 200 |
| 10 | contig_1374 | (AGACCCT)n | hepta | 1,247 | 1,443 | 197 |
| 11 | contig_1879 | (AGGGCTC)n | hepta | 66,884 | 67,075 | 186 |
| 12 | contig_469 | (ATGGGCT)n | hepta | 376,522 | 376,703 | 182 |
| 13 | contig_65 | (CATACAA)n | hepta | 55,721 | 55,893 | 177 |
| 14 | contig_749 | (TCCGGAG)n | hepta | 206,925 | 207,093 | 166 |
| 15 | contig_1119 | (CTGCTCC)n | hepta | 210,554 | 210,719 | 162 |
| 16 | contig_111 | (TCCGACA)n | hepta | 184,518 | 184,650 | 154 |
| 17 | contig_2550 | (TTCCTCG)n | hepta | 1,601 | 1,754 | 154 |
| 18 | contig_1485 | (CAGAGCC)n | hepta | 1,262,462 | 1,262,611 | 150 |
| 19 | contig_8930 | (GTTTAAG)n | hepta | 105,811 | 105,961 | 146 |
| 20 | contig_1419 | (TGCCGGC)n | hepta | 150,723 | 150,869 | 145 |
| 21 | contig_2527 | (CGCCTGA)n | hepta | 87,059 | 87,191 | 139 |
| 22 | contig_2073 | (TATGTTA)n | hepta | 45,233 | 45,373 | 137 |
| 23 | contig_737 | (AAGCAAT)n | hepta | 6,932 | 7,071 | 134 |
| 24 | contig_3531 | (AAGGTTT)n | hepta | 109,441 | 109,576 | 133 |
| 25 | contig_8761 | (ACTAGCT)n | hepta | 2,643 | 2,773 | 132 |
| 26 | contig_3660 | (GCGTGCT)n | hepta | 174,080 | 174,180 | 128 |
| 27 | contig_779 | (TAGCACA)n | hepta | 603,552 | 603,677 | 128 |
| 28 | contig_135 | (TAACTTG)n | hepta | 326,515 | 326,635 | 125 |
| 29 | contig_1003 | (GTGGAAG)n | hepta | 5,422 | 5,544 | 119 |
| 30 | contig_3267 | (TTCTCAA)n | hepta | 43,227 | 43,335 | 115 |
| 31 | contig_2939 | (CCGGAGG)n | hepta | 69,511 | 69,627 | 112 |
| 32 | contig_836 | (CTCAACA)n | hepta | 249,536 | 249,642 | 110 |
| 33 | contig_422 | (CCCCGAG)n | hepta | 287,529 | 287,635 | 108 |
| 34 | contig_4880 | (TTAATGT)n | hepta | 38,070 | 38,177 | 106 |
| 35 | contig_1157 | (GATCTTG)n | hepta | 387,365 | 387,474 | 105 |
| 36 | contig_2842 | (GGCGGTT)n | hepta | 94,531 | 94,636 | 104 |
| 37 | contig_5593 | (TCTCTTA)n | hepta | 16,636 | 16,741 | 104 |
| 38 | contig_3011 | (GCGAGTG)n | hepta | 12,163 | 12,268 | 103 |
| 39 | contig_3225 | (TTTCATC)n | hepta | 204,660 | 204,767 | 103 |
| 40 | contig_2135 | (GCCGCCAA)n | octa | 33,373 | 33,605 | 249 |
| 41 | contig_3596 | (CCCGCCGG)n | octa | 199,857 | 200,074 | 226 |
| 42 | contig_3263 | (TTTATTAT)n | octa | 181,244 | 181,447 | 205 |
| 43 | contig_1430 | (GCATCGCC)n | octa | 46,401 | 46,529 | 137 |
| 44 | contig_2693 | (CGCCCGCT)n | octa | 418,746 | 418,850 | 119 |
| 45 | contig_1763 | (GTATGGA)n | octa | 111,986 | 112,100 | 119 |
| 46 | contig_2308 | (GTTTGTGA)n | octa | 156,752 | 156,863 | 116 |
| 47 | contig_1530 | (TTTTATCA)n | octa | 150,870 | 150,880 | 108 |
| 48 | contig_316 | (CGCGGCGC)n | octa | 326,992 | 327,093 | 105 |
| 49 | contig_3038 | (AGTTCACAC)n | nona | 162,707 | 162,927 | 215 |
| 50 | contig_5760 | (GCTATGTGA)n | nona | 103,511 | 103,659 | 144 |
| 51 | contig_2775 | (AACTGTGTG)n | nona | 55,320 | 55,440 | 118 |
| 52 | contig_700 | (CACATAGCT)n | nona | 221,391 | 221,504 | 106 |
| 53 | contig_177 | (TGTGAACTG)n | nona | 719,771 | 719,877 | 104 |
| 54 | contig_836 | (AGGTTCTGGA)n | deca | 465,813 | 465,936 | 127 |
| 55 | contig_2705 | (AACTAACCCT)n | deca | 28,052 | 28,166 | 112 |
| 56 | contig_1324 | (TCCAGAACCT)n | deca | 707,617 | 707,720 | 109 |
| 57 | contig_3610 | (GTTCCGGAAG)n | deca | 532,860 | 532,965 | 104 |
| 58 | contig_645 | (TTTTTCTGAA)n | deca | 342,606 | 342,705 | 104 |

Supplementary Table S4. List of samples used in the molecular analyses.

| Taxon | Voucher No in KRA/ALTB | Locality | Latitude | Longitude | Altitude | Data | Collector |
| --- | --- | --- | --- | --- | --- | --- | --- |
| *S. capillata* | 0496240 | Kyrgyzstan,  central Tian Shan,  ca. 47 km NNE of Chaek | N 42°4'41.29" | E 75°3'12.43" | 2387 m | 07.07.2018 | M.Nobis,  E.Klichowska,  A.Wróbel, A.Nowak |
| *S. richteriana* | 003756 | Kazakhstan,  Moiynkum distr.,  between Kashkanteniz and Mynaral | N 45°30'38.88" | E 73°29'38.34" | 451 m | 22.05.2014 | M.Nobis,  P.Gudkova |
| *S. lessingiana* | 003728 | Kazakhstan,  Zhambyl distr.,  10 km E of Targap | N 43°19'21" | E 75°55'50" | 755 m | 18.05.2014 | M.Nobis,  P.Gudkova |
| *S. heptapotamica* | 003747 | Kazakhstan,  Kerbulak distr.,  2 km NE of Karlygash | N 44°13'40.4" | E 77°42'27.6" | 966 m | 22.05.2014 | M.Nobis,  P.Gudkova |
| *S. korshinskyi* | 001767 | Russia,  Altayskiy kray, Krasnoshchyokovsky distr.,  between Kurya and Krasnoshchekovo | N 51°36'59.88" | E 82°33'59.7" | 277 m | 01.06.2016 | E.Punina |

Supplementary Table S5**.** Species names and GenBank accession numbers for the reference mitochondrial (mt) and chloroplast (cp) genomes used in this study.

| Taxon | Type | Sequence length (bp) | GB accession number |
| --- | --- | --- | --- |
| *Tripsacum dactyloides* | mt | 704,100 | NC_008362.1 |
| *Hordeum vulgare* | mt | 525,599 | AP017300.1 |
| *Zea mays* | mt | 569,630 | NC_007982.1 |
| *Triticum aestivum* | mt | 452,526 | MH051716.1 |
| *Eleusine indica* | mt | 520,691 | NC_040989.1 |
| *Sorghum bicolor* | mt | 468,628 | NC_008360.1 |
| *Oryza sativa* | mt | 637,692 | JF281153.1 |
| *Aegilops speltoides* | mt | 476,091 | NC_022666.1 |
| *Alloteropsis semialata* | mt | 442,063 | MH644808.1 |
| *Lolium perenne* | mt | 678,580 | JX999996.1 |
| *Saccharum officinarum*, chromosome 1 | mt | 300,784 | NC_031164.1 |
| *Saccharum officinarum*, chromosome 2 | mt | 144,698 | LC107875.1 |
| *Stipa arabica* | cp | 137,757 | NC_037024 |
| *Stipa borysthenica* | cp | 137,825 | NC_037025 |
| *Stipa capillata* | cp | 137,830 | MG052598 |
| *Stipa capillata* | cp | 137,835 | MG052599 |
| *Stipa caucasica* | cp | 137,798 | MG052600 |
| *Stipa heptapotamica* | cp | 137,829 | MH918066 |
| *Stipa hohenackeriana* | cp | 137,753 | NC_037028 |
| *Stipa hymenoides* | cp | 137,742 | NC_027464 |
| *Stipa jagnobica* | cp | 137,827 | MG052604 |
| *Stipa lessingiana* | cp | 137,829 | NC_037030 |
| *Stipa lipskyi* | cp | 137,854 | KT692644 |
| *Stipa magnifica* | cp | 137,848 | MG052606 |
| *Stipa narynica* | cp | 137,854 | MG052607 |
| *Stipa orientalis* | cp | 137,822 | NC_037033 |
| *Stipa ovczinnikovii* | cp | 137,874 | NC_037034 |
| *Stipa pennata* | cp | 137,825 | MG052610 |
| *Stipa purpurea* | cp | 137,370 | KT983629 |
| *Stipa richteriana* | cp | 137,831 | MG052612 |
| *Stipa roylei* | cp | 137,606 | MT094322 |
| *Stipa tianschanica* | cp | 137,847 | MG052613 |
| *Stipa x alaica* | cp | 137,850 | MG052614 |
| *Stipa x brevicallosa* | cp | 137,850 | MG052615 |
| *Stipa zalesskii* | cp | 137,836 | MG052616 |

Supplementary information S1. CTAB large-scale DNA extraction protocol.

**Before extraction:**

1) Prepare 5 ml of fresh (no more than 1 day old) CTAB buffer:

• 100 mM Tris-HCl pH 8.0 = Add 500 uL 1M Tris-HCl.

• 20 mM EDTA = Add 200 uL 0.5N EDTA.

• 1.42 M NaCL = Add 415 mg NaCl.

• 55 mM CTAB = Add 100 mg CTAB.

• 0.5 mM PVP = Add 100 mg PVP.

• Add 4.3 ml water for 5.0 mL total volume.

• Directly before use, add 4 uL βME to each 2 mL of CTAB buffer.

2) Prepare:

• 24:1 chloroform:isoamyl alcohol.

• Fresh 70% ethanol.

• Isopropanol for precipitation.

3) Place metal spatulas with dry ice to freeze. Be sure that 37°C and 65°C water baths are prepared.

**Extraction steps:**

1) Grind ~0.2 g tissue in pre-chilled mortar and pestle under liquid nitrogen until it is a fine powder. With a cold spatula, quickly transfer the powder to a 15 ml falcon tube.

2) Add 2 ml of CTAB + βME to the tube before the tissue thaws and invert repeatedly (do not vortex).

3) Place sample in 65°C water bath and incubate for 20 minutes, inverting every 5 minutes to insure tissue is completely homogenized and mixed with CTAB buffer.

4) Centrifuge at > 4000g for 15 minutes to pellet plant tissue, and transfer supernatant to new 15 ml tube.

5) To supernatant in new tube, add 2 ml 24:1 chloroform:isoamyl alcohol. Mix and invert continuously for 5-10 minutes.

6) Centrifuge at > 4000g for 15 minutes to create pellet. Being careful to not disrupt interface layer, transfer supernatant to new 15 ml tube.

7) Add Qiagen RNase A to the transferred supernatant to achieve 1.8% of total volume. Incubate at 37°C for 15-30 minutes (with occasional mixing).

8) After RNase digestion, add 2 ml 24:1 chloroform:isoamyl alcohol. Mix and invert continuously for 5-10 minutes.

9) Centrifuge at > 4000g for 15 minutes to create pellet. Being careful to not disrupt interface layer, transfer supernatant to a new 15 ml tube.

10) To precipitate DNA, add 2 ml of room temperature isopropanol to the surface of the supernatant and gradually rock to mix. Continue mixing gently for 5-10 minutes, at which point you should see the DNA precipitate out of solution. You can continue to precipitate DNA by placing at -20°C for 1 hour, but most of the high molecular weight DNA precipitates in the first few minutes.

11) After precipitation, centrifuge at > 4000g for 15 minutes to pellet DNA. Pour out the alcohol carefully, being sure the white pellet remains at the bottom of the tube.

12) Add 5 ml 70% ethanol, mix by inverting the tube, spin for 5 min, and pour out the alcohol.

13) Repeat the 70% ethanol wash.

14) Perform a final brief spin to collect residual ethanol. Remove this carefully with a p20 pipet.

15) Air dry the pellet for no more than 5-10 minutes on the bench top.

16) Resuspend the pellet in 200 uL of EB buffer on the bench for 1-2 hours, then overnight at 4°C.

Supplementary information S2. Software tools, versions, settings and parameters used in the study.

**(1) AliView:** version 1.26, default parameters;

**(2) Assembly-stats:** version 1.0.1, default parameters;

**(3) Augustus:** versions 3.2.3, 3.3.3, 3.3.4, default parameters;

**(4) BEAST2:** version 2.6.3, default parameters;

**(5) BLAST:** version 2.10.0, default parameters;

**(6) BUSCO:** version 4.0.6, dataset poales_odb10, default parameters;

**(7) Canu v.2.0:** version 2.0, parameters: (minReadLength=5000 minOverlapLength=5000 genomeSize=0.01m);

**(8) Circlator:** version 1.5.5, default parameters;

**(9) Cp-hap:** default parameters;

**(10) FALCON:** version 0.2.5, default parameters;

**(11) Flye:** version 2.4 for the nuclear genome assembly, parameter: (--genome-size 600m);

**(12) Flye:** version 2.7.1-b1590 for the mitochondrial and chloroplast genome assemblies, parameter for the first run: (--genome-size 0.55m); parameters for the second run: (--genome-size 0.55m --trestle --min-overlap 10000);

**(13) Krait:** version 1.3.3, default parameters;

**(14) MAFFT:** version 7.471, parameters: (--ep 0 --op 12 --lexp 3 --genafpair --maxiterate 1000);

**(15) Minimap2:** version 2.17-r941, default parameters;

**(16) Purge Haplotigs:** version 1.1.1, default parameters;

**(17) Qualimap:** version 2.2.2, default parameters;

**(18) RaGOO:** version 1.1, default parameters;

**(19) RepeatMasker:** version 4.1.0, custom library, default parameters;

**(20) RepeatModeler:** version 2.0.1, default parameters;

**(21) Samtools:** version 1.9**,** parameters: (view -F 4 -q 20);

**(22) SequelQC:** version 1.1.0, parameters: (-k -p a);

**(23) SPAdes:** version 3.14.1, parameter: (-k 95);

**(24) Unicycler:** version 0.4.8, default parameters.
